# Morphological and molecular characterisation of *Longidorus pauli* (Nematoda: Longidoridae), first report from Greece

**DOI:** 10.21307/jofnem-2021-034

**Published:** 2021-03-20

**Authors:** Emmanuel A. Tzortzakakis, Ilenia Clavero-Camacho, Carolina Cantalapiedra-Navarrete, Parthenopi Ralli, Juan E. Palomares-Rius, Pablo Castillo, Antonio Archidona-Yuste

**Affiliations:** 1 Department of Viticulture, Vegetable Crops, Floriculture and Plant Protection, Institute of Olive Tree, Subtropical Crops and Viticulture, N.AG.RE.F., Hellenic Agricultural Organization – DIMITRA, 32A Kastorias street, Mesa Katsabas, 71307, Heraklion, Crete, Greece; 2 Institute for Sustainable Agriculture (IAS), CSIC, Avenida Menéndez Pidal s/n, 14004 Córdoba, Campus de Excelencia Internacional Agroalimentario, ceiA3, Spain; 3 Institute of Plant Breeding and Genetic Resources, N.AG.RE.F., Hellenic Agricultural Organization – DIMITRA, Thermi, Thessaloniki, Greece; 4 Department of Ecological Modelling, Helmholtz Centre for Environmental Research – UFZ, Permoserstrasse 15, 04318 Leipzig, Germany

**Keywords:** Cytochrome oxidase c subunit 1, D2-D3 of 28S rRNA, Description, ITS1 rRNA, *Longidorus*, *L. pauli*, *L. pisi*, Needle nematodes, Taxonomy

## Abstract

Sampling for needle nematodes was carried out in a grapevine area in Thessaloniki, North Greece and two nematode species of *Longidorus* (*L. pauli* and *L. pisi*) were collected. Nematodes were extracted from 500 cm^3^ of soil by modified sieving and decanting method, processed to glycerol and mounted on permanent slides, and subsequently identified morphologically and molecularly. Nematode DNA was extracted from single individuals and PCR assays were conducted to amplify D2-D3 expansion segments of 28S rRNA, ITS1 rRNA, and partial mitochondrial coxI regions. Morphology and morphometry data obtained from these populations were consistent with *L. pauli* and *L. pisi* identifications. To our knowledge, this is the first report of *L. pauli* for Greece, and the second world report after the original description from Idleb, Syria, extending the geographical distribution of this species in the Mediterranean Basin.

Needle nematodes are polyphagous root ectoparasites of a wide range of economically important plants by directly feeding on root cells. Some species of this genus are economically important pests of agricultural plants, and others are proved to transmit nepoviruses (Taylor and Brown, 1997). The genus *Longidorus* consists of more than 160 valid species (Archidona-Yuste et al., 2016; Cai et al., 2020), but currently only 12 have been reported from Greece. Seven of these have been molecularly identified *Longidorus closelongatus* (Stoyanov, 1964), *Longidorus cretensis* (Tzortzakakis et al., 2001), *Longidorus euonymus* (Mali and Hooper, 1974), *Longidorus iranicus* ( =  *moesicus*) (Sturhan and Barooti, 1983), *Longidorus orientalis* (Loof, 1982), *Longidorus pisi* ( =  *latocephalus*) (Edward et al., 1964), and *Longidorus pseudoelongatus* (Altherr, 1976; He et al., 2005, Tzortzakakis et al., 2014, 2017). The five remaining species *L. africanus* (Merny, 1966), *L. elongatus* (de Man, 1876; Micoletzky, 1922) *L. fasciatus* (Roca and Lamberti, 1981), *L. intermedius* (Kozlowska and Seinhorst, 1979), and *L. proximus* (Sturhan and Argo, 1983) lack of molecular characterization (Tzortzakakis et al., 2008). During a recent nematode sampling from a grapevine area in Northern Greece, two needle nematode populations were detected resembling *L. pauli* (Lamberti et al., 1999) and *L. pisi*. *Longidorus pauli* was previously reported only from original description on fig tree at Idleb, Syria (Lamberti et al., 1999). Therefore, the objective of the present study was to provide an accurate identification of *Longidorus* species detected in North Greece by an integrative approach of morphological and molecular characterization by using the D2-D3 expansion segments of 28S rRNA, ITS1 rRNA, and partial mitochondrial coxI regions.

## Materials and methods

### Nematode samples and morphological study

Soil samples were collected at a depth of 20 to 40 cm from the rhizosphere of a grapevine grafted on 1103-Paulsen of the Institute of Plant Breeding and Genetic Resources, Thermi, Thessaloniki, Greece. Nematodes were extracted from soil by modified sieving and decanting method (Brown and Boag, 1988). Extracted specimens were heat killed, fixed in TAF, processed to glycerol by a slow evaporation method, and mounted on permanent slides (Hooper, 1986). The light micrographs and measurements of nematode populations including the main diagnostic characteristics (i.e., de Man indices, body length, odontostyle length, lip region, tail shape, amphid shape, and oral aperture-guiding ring) were performed using a Leica DM6 compound microscope with a Leica DFC7000 T digital camera. All abbreviations were used as defined in Jairajpuri and Ahmad (1992).

### Molecular characterization

For molecular analyses, and in order to avoid mistakes in case of mixed populations in the same sample, single specimens from the sample were temporarily mounted in a drop of 1 M NaCl containing glass beads (to avoid nematode crushing/damaging specimens) to ensure specimens conformed with the target population. All necessary morphological and morphometric data were recorded. This was followed by DNA extraction from single individuals as described by Archidona-Yuste et al. (2016). The D2-D3 segments were amplified using the D2A (5´-ACAAGTACCGTGAGGGAAAGTTG-3´) and D3B (5´-TCGGAAGGAACCAGCTACTA-3´) primers (De Ley et al., 1999). The Internal Transcribed Spacer region 1 (ITS1) separating the 18S rRNA gene from the 5.8S rRNA gene was amplified using forward primer 18S (5´-TTGATTACGTCCCTGCCCTTT-3´) (Vrain et al., 1992) and reverse primer rDNA1 5.8S (5´-ACGAGCCGAGTGATCCACCG-3´) (Cherry et al., 1997). Finally, the portion of the coxI gene was amplified as described by Lazarova et al. (2006) using the primers COIF (5´-GATTTTTTGGKCATCCWGARG-3´) and COIR (5´-CWACATAATAAGTATCATG-3´).

All PCR assays were done according to the conditions described by Archidona-Yuste et al. (2016). Then, the amplified PCR products were purified using ExoSAP-IT (Affimetrix, USB products. COUNTRY) and used for direct sequencing on a DNA multicapillary sequencer (Model 3130XL genetic analyzer; Applied Biosystems, Foster City, CA, USA), using the BigDye Terminator Sequencing Kit V.3.1 (Applied Biosystems, Foster City, CA, USA), at the Stab Vida sequencing facilities (Caparica, Portugal). The newly obtained sequences were submitted to the GenBank database under the accession numbers indicated on the phylogenetic trees. This population of *Longidorus* is proposed here as standard and reference population for *L. pauli* until topotype material becomes available and molecularly characterized. Voucher specimens of this described species have been deposited in the nematode collection of Institute for Sustainable Agriculture, IAS-CSIC, Córdoba, Spain.

### Phylogenetic analyses

D2-D3 expansion segments of 28S rRNA, ITS1 rRNA, and coxI mtDNA sequences of the unidentified *Longidorus* species population were obtained in this study. These sequences, and other sequences from species of *Longidorus* from GenBank, were used for phylogenetic analyses. Outgroup taxa for each dataset were chosen following previously published studies (Archidona-Yuste et al., 2019; Cai et al., 2020; He et al., 2005; Holterman et al., 2006). Multiple sequence alignments of the different genes were made using the FFT-NS-2 algorithm of MAFFT V.7.450 (Katoh et al., 2019). Sequence alignments were manually visualized using BioEdit (Hall, 1999) and edited by Gblocks ver. 0.91b (Castresana, 2000) in the Castresana Laboratory server (http://molevol.cmima.csic.es/castresana/Gblocks_server.html) using options for a less stringent selection (minimum number of sequences for a conserved or a flanking position: 50% of the number of sequences + 1; maximum number of contiguous non-conserved positions: 8; minimum length of a block: 5; allowed gap positions: with half). Phylogenetic analyses of the sequence datasets were based on Bayesian inference (BI) using MrBayes 3.1.2 (Ronquist and Huelsenbeck, 2003). The best-fit model of DNA evolution was obtained using JModelTest V.2.1.7 (Darriba et al., 2012) with the Akaike information criterion (AIC). The best-fit model, the base frequency, the proportion of invariable sites, and the gamma distribution shape parameters and substitution rates in the AIC were then used in MrBayes for the phylogenetic analyses. The general time-reversible model with invariable sites and a gamma-shaped distribution (GTR + I + G) for the D2-D3 segments of 28S rRNA and the partial coxI gene, and the transitional model and a gamma-shaped distribution (TIM3+G) model for ITS1 rRNA were analyzed with four chains for 2 × 10^6^ generations, respectively. A combined analysis of the three ribosomal genes was not undertaken due to some sequences not being available for all species. The Markov chains were sampled at intervals of 100 generations and two runs were conducted for each analysis. After discarding burn-in samples of 30% and evaluating convergence, the remaining samples were retained for in-depth analyses. The topologies were used to generate a 50% majority rule consensus tree. Posterior probabilities (PP) were given on appropriate clades. Trees from all analyses were visualized using FigTree software version v.1.42 (Rambaut, 2014).

## Results and discussion

Soil samples from grapevine at Thessaloniki, North Greece yielded two *Longidorus* populations, including a moderately abundant population (5-10 needle nematodes/500 cm^3^ of soil) resembling *L. pauli* and two juvenile specimens of *L. pisi* that were confirmed by 28S rRNA, which were 100% coincident with a population from Bulgaria (LR032064) and 99% (AY601569, differing in 1 nucleotide) from a Greek population (He et al., 2005; Peneva et al., 2013). Since in recent studies, this species has been molecularly characterized by D2-D3 28S rRNA or the coxI gene for populations from Greece, South Africa, and Iran (He et al., 2005; Palomares-Rius et al., 2017; Pedram et al., 2008), no further studies were developed on this new Greek population.

### Systematics

**Figure 1:**
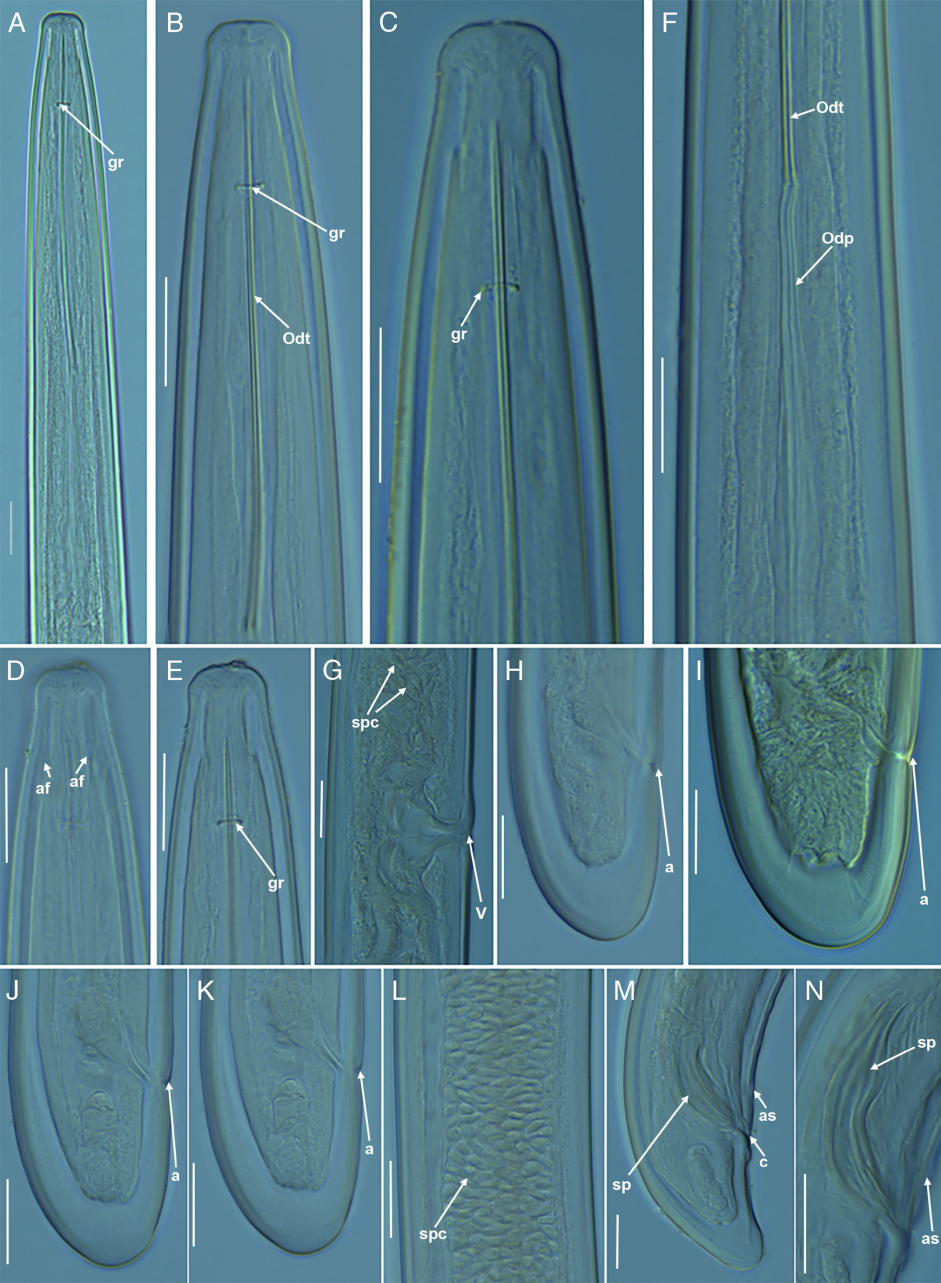
Light micrographs of *Longidorus pauli* (Lamberti et al., 1999) from Greece (A-N). A-E: Female anterior regions. F: Detail of base of odontostyle and odontophore, G: Vulval region showing sperm cells in the uterus, H-K: Female tail regions, L: Detail of sperm cells in male genital tract, M: Male tail region, N: Detail of spicules. a = anus; af = amphidial fovea; as = adanal supplement; c = cloaca; gr = guiding ring; Odt = odontostyle; Odp = odontophore; sp = spicules; spc = sperm cells; V = vulva. (Scale bars: A-N = 20 µm).

*Longidorus pauli* (Lamberti et al., 1999) (Fig. 1 and Table 1).

**Table 1.** Morphometrics of *Longidorus pauli* (Lamberti et al., 1999) from Greece.

	Thessaloniki, Greece		Idleb, Syria (Lamberti et al., 1999)	
Character^a^	Females	Males	Females	Males
*n*	8	5	20	12
*L* (mm)	7.6 ± 0.66 (6.62-8.51)	7.19 ± 0.27 (6.66-8.02)	7.6 ± 0.51 (6.5-8.6)	7.7 ± 0.64 (6.8-8.7)
*a*	147.9 ± 15.3 (125.0-168.8)	150.1 ± 8.9 (138.3-162.5)	131.6 ± 8.5 (120.3-143.5)	139.9 ± 7.9 (130.8-154.8)
*b*	16.0 ± 1.7 (13.9-19.0)	16.5 ± 1.3 (14.8-17.8)	16.9 ± 1.6 (14.5-19.7)	16.1 ± 1.8 (12.8-19.5)
*c*	173.1 ± 29.0 (126.9-224.0)	158.9 ± 12.3 (146.1-175.3)	200.7 ± 17.9 (163.5-220.0)	181.9 ± 11.7 (166.0-197.8)
*c’*	1.1 ± 0.1 (1.0-1.3)	1.2 ± 0.1 (1.0-1.3)	0.9 ± 0.1 (0.8-1.0)	0.97 ± 0.05 (0.9-1.0)
*d* ^b^	2.1 ± 0.2 (1.9-2.4)	2.3 ± 0.2 (2.0-2.6)	–	–
*d’* ^*c*^	1.6 ± 0.1 (1.4-1.8)	1.5 ± 0.1 (1.4-1.6)	–	–
V/Spicules length	52.1 ± 2.2 (49.4-56.0)	57.6 ± 0.4 (54.0-60.0)	51 ± 1.5 (49.0-54.0)	64.6 ± 2.5 (61.0-69.0)
Odontostyle length	117.4 ± 5.5 (112.0-126.0)	113.6 ± 7.4 (103.0-120.0)	109.4 ± 3.6 (102.0-118.3)	109.0 ± 4.7 (101.5-117.7)
Odontophore length	63.5 ± 2.2 (61.0-67.0)	63.4 ± 3.3 (59.0.68.0)	61.2 ± 2.5 (56.0-64.0)	63.0 ± 1.8 (61.0-66.5)
Total stylet length	180.9 ± 6.8 (173.0-192.0)	177.0 ± 6.7 (167.0-184.0)	–	–
Anterior end to guide ring	31.6 ± 1.9 (28.0-33.5)	33.2 ± 1.3 (32.0-35.0)	30.6 ± 2.1 (27.2-35.8)	30.4 ± 1.6 (27.7-32.9)
Tail length	42.9 ± 2.1 (38.0-45.0)	46.6 ± 1.3 (44.0-49.0)	37.8 ± 3.2 (31.5-45.0)	42.4 ± 3.3 (36.4-46.3)
Hyaline part of tail length	16.4 ± 1.5 (14.0-18.5)	14.3 ± 2.0 (12.5-17.0)	13.1 ± 1.1 (10.5-15.4)	11.5 ± 1.6 (10.0-14.9)
Body width at level of				
lip region	14.9 ± 0.7 (13.5-16.0)	14.8 ± 1.0 (13.5-16.0)	15.2 ± 0.9 (14.0-17.0)	14.2 ± 0.5 (13.9-15.0)
guide ring	23.1 ± 1.5 (21-24.5)	22.6 ± 1.5 (20.0-23.5)	23.5 ± 1.1 (21.0-25.0)	21.8 ± 1.0 (19.6-23.0)
anus	38.8 ± 1.3 (37.0-41.5)	36.6 ± 1.5 (32.0-39.0)	41.4 ± 2.4 (36.6-44.6)	42.6 ± 2.3 (37.6-45.7)

**Notes:** Measurements in µm, at exception of *L* in mm. ^a^Abbreviations are defined in Jairajpuri and Ahmad (1992); ^b^ *d* = anterior to guide ring/body width at lip region (Brown et al., 1994); ^c^ *d’* = body width at guide ring/body width at lip region (Brown et al., 1994).

### Description

Female: body ventrally curved in a close C-shaped to single spiral when killed by gentle heat with greater curvature in the posterior half more pronounced in the case of male. Moderate long body length (6.6-8.5 mm in female; and 6.6-8.0 mm in male). Cuticle (3.0-4.0) µm thick at mid-body. Lip region rounded and set off by a slight depression from the rest of body, anteriorly slightly rounded to flattened (Fig. 1). Amphidial pouch slightly asymmetrically bilobed. Guiding ring single, located 1.9 to 2.4 times lip region diameter from anterior end. Odontostyle 1.8 to 2.0 times as long as odontophore; odontophore well developed, with slight basal swellings. Esophagus extending to a terminal esophageal bulb (107.0-127.0) μm long), with dorsal (DN) gland nucleus and ventrosublateral (SVN) gland nuclei separately located at (20.6-28.9)% and (51.4-57.0)% of distance from anterior end of esophageal bulb, respectively. Glandularium (97.0-118.0) μm long. Cardia conoid-rounded. Vulva located about mid-body or slightly posterior (49.4-56.0%). Vagina (9.0-12.0) µm wide, ovijector (23.0-34.0) µm wide. Genital tract amphidelphic, anterior and posterior genital branches equally developed, 414 to 772 and 407 to 647 µm long, respectively. Rectum 23 to 25 µm long. Sperm cells (5.0-6.0) μm long, frequently detected in both genital branches. Tail conoid-rounded with bluntly rounded terminus.

Male: morphologically similar to female and common. Testes paired, full of oblong sperm cells. Adanal supplements paired, at (10.5-14.5) µm from anus, preceded anteriorly by a row of 9 to 15 irregularly spaced ventromedians supplements. Spicules paired, robust and ventrally curved, approximately 1.1 to 1.3 times larger than tail length. Lateral guiding pieces with a curved proximal end. According to the polytomous key Chen et al. (1997), supplement by Loof and Chen (1999), and the addition of some characters by Peneva et al. (2013), the Greek population has the following codes (codes in parentheses are exceptions): A4 – B2(3) – C2(3) – D3 – E2(3) – F4 – G3 – H1 – I2 – J1 – K7.

### Remarks

The Greek population of *L. pauli* was collected from the rhizosphere of grapevine at Thermi, Thessaloniki, Greece with a nematode density of 5 to 10 nematodes/500 cm^3^ soil. Up to our knowledge, this is the first report of this species from Greece and the second after original description from Syria by Lamberti et al. (1999). Morphology and morphometrics of Greek population agree with those of the type population of this species (Table 1). The main differences between Greek population of *L. pauli* and original population of this species are: a ratio ((125.0-168.8) vs (120.3-143.5)), *c´* ratio ((1.0-1.3) vs (0.8-1.0)), odontostyle length ((112.0-126.0) vs (102.0-118.3) µm), and spicules length ((54.0-60.0) vs (61.0-69.0) µm). These small morphometrics differences detected may be due to geographical intraspecific variability of them.

This species is morphological- and morphometrically close *L. closelongatus*, *L. pseudoelongatus*, *L. apulus* (Lamberti and Bleve Zacheo, 1977), and *L. apuloides* (Roca, 1996), from which can be separated by (i) *L. closelongatus*: longer body length ((6.6-8.5) vs (5.3-7.3) mm) and lower *c´* ratio ((1.0-1.3) vs (1.3-1.5)); (ii) *L. pseudoelongatus*: longer body length ((6.6-8.5) vs (5.1-5.6) mm), higher a ratio ((125.0-168.8) vs (73.0-110.0)), and slightly higher *V* ratio ((49.4-56.0) vs (45.0-48.0)); (iii) *L. apulus*: longer odontostyle length ((112.0-126.0) vs (91.0-112.0) µm); and (iv) *L. apuloides* slightly shorter body length ((6.6-8.5) vs (7.4-10.2) mm) and spicules length ((54.0-60.0) vs (52.5-86.0) µm). From all of them can be also separated by molecular markers 28S rRNA, ITS rRNA, and coxI regions, except for *L. apuloides* which has not been molecularly characterized yet.

Molecular characterization and phylogeny of *Longidorus pauli* Amplification and sequencing of the D2-D3 expansion domains of 28S rRNA, ITS1 rRNA, and partial coxI genes yielded sequences sizes of *ca* 750 bp, 1,000 bp, and 400 bp, respectively, based on gel electrophoresis. Five new D2-D3 of 28S rRNA gene sequences of *L. pauli* were obtained in the present study (MW598384-MW598388) and showed a low intraspecific variability with 0 to 1 different nucleotides and 0 indels (99% similarity). The D2-D3 for *L. pauli*. differed from the closest related species, *L. proximus* (MK894275) by 19 nucleotides and 0 indel (98% similarity), *L. iranicus* (MK894273) by 17 nucleotides and two indels (98% similarity), *L. closelongatus* (KJ802866) by 25 nucleotides and 0 indel (97% similarity), and from *L. cretensis* (KJ802868) by 27 nucleotides and 0 indel (96% similarity).

The ITS1 region also showed a low intraspecific variability by 0 to 2 nucleotides and 1 indel (99% similarity). ITS1 for *L. pauli* (MW598390-MW598392) showed low similarity with all the ITS1 sequences of *Longidorus* spp. deposited in NCBI, including the most similar species, *L. cretensis* (KJ802892), *L. iranicus* (KP222295), and *L. closelongatus* (KJ802891), by 159 to 179 different nucleotides and 58 indels (83-85% similarity).

The four new coxI sequences for *L. pauli* showed moderate intraspecific variability by 20 to 23 nucleotides and 0 indel (94% similarity). coxI for *L. pauli* (MW598436-MW598439) showed low similarity with all the coxI sequences of *Longidorus* spp. deposited in NCBI, including the most similar species by 70, 76, 81 and 77 nucleotides (78-80% similarity) and 0 to 2 indels from the closest related species, *L. pseudoelongatus* (KY816699), *L. iranicus* (KY816677), *L. pini* (MH454070) and *L. cretensis* (KY816670), respectively.

**Figure 2:**
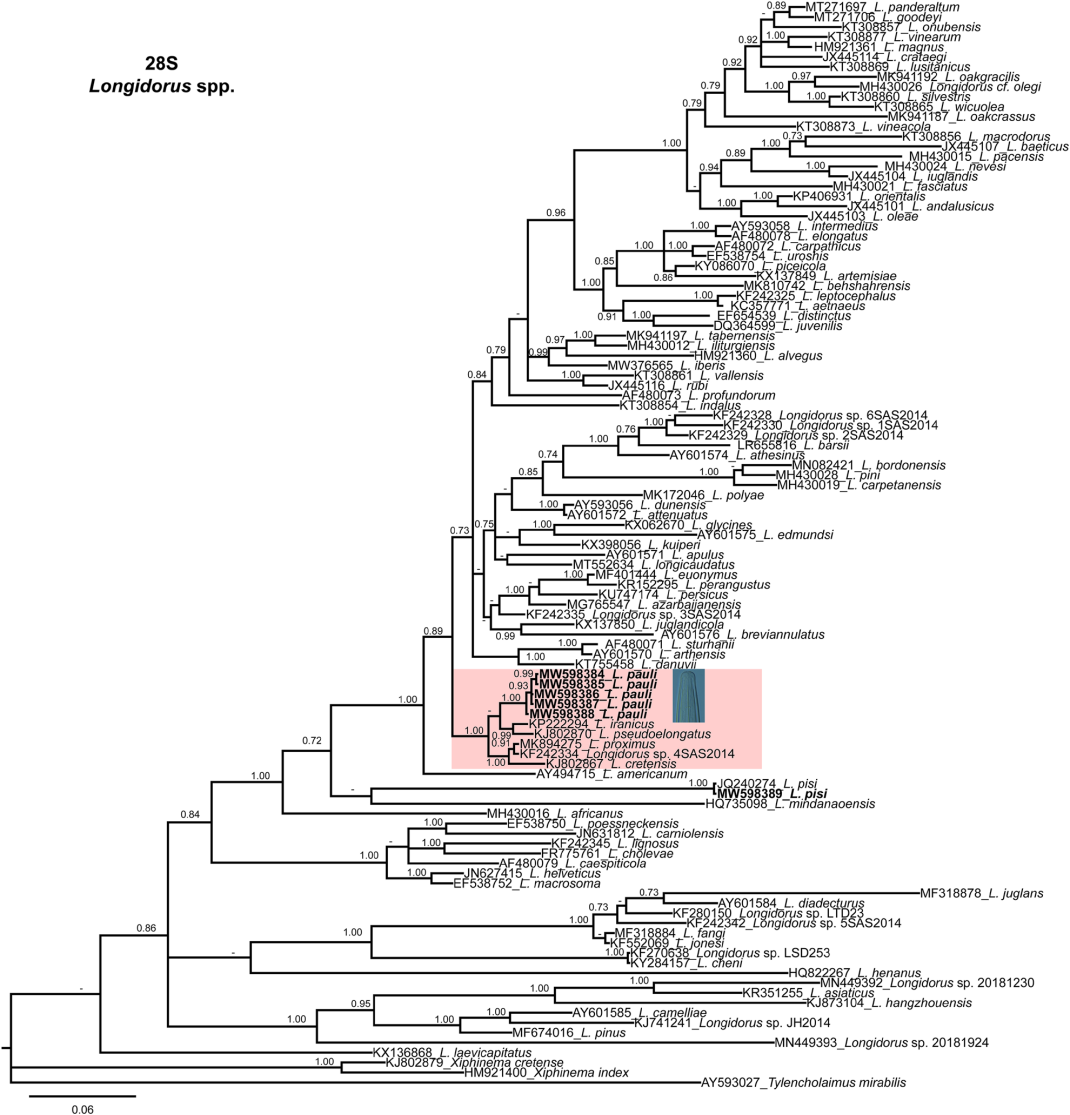
Phylogenetic relationships of *Longidorus pauli* (Lamberti et al., 1999) within the genus *Longidorus*. Bayesian 50% majority rule consensus tree as inferred from D2 and D3 expansion domains of 28S rRNA sequence alignment under the general time-reversible model of sequence evolution with correction for invariable sites and a gamma-shaped distribution (GTR + I + G). Posterior probabilities more than 0.70 are given for appropriate clades. Newly obtained sequences in this study are shown in bold. Scale bar = expected changes per site.

**Figure 3:**
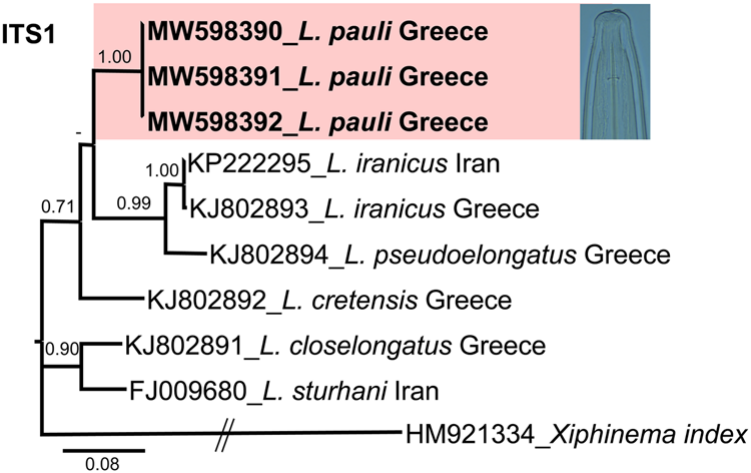
Phylogenetic relationships of *Longidorus pauli* (Lamberti et al., 1999) from Greece within the genus *Longidorus*. Bayesian 50% majority rule consensus tree as inferred from ITS1 rRNA sequence alignment under the TIM3 + G model. Posterior probabilities more than 0.70 are given for appropriate clades. Newly obtained sequences in this study are shown in bold. Scale bar = expected changes per site.

**Figure 4:**
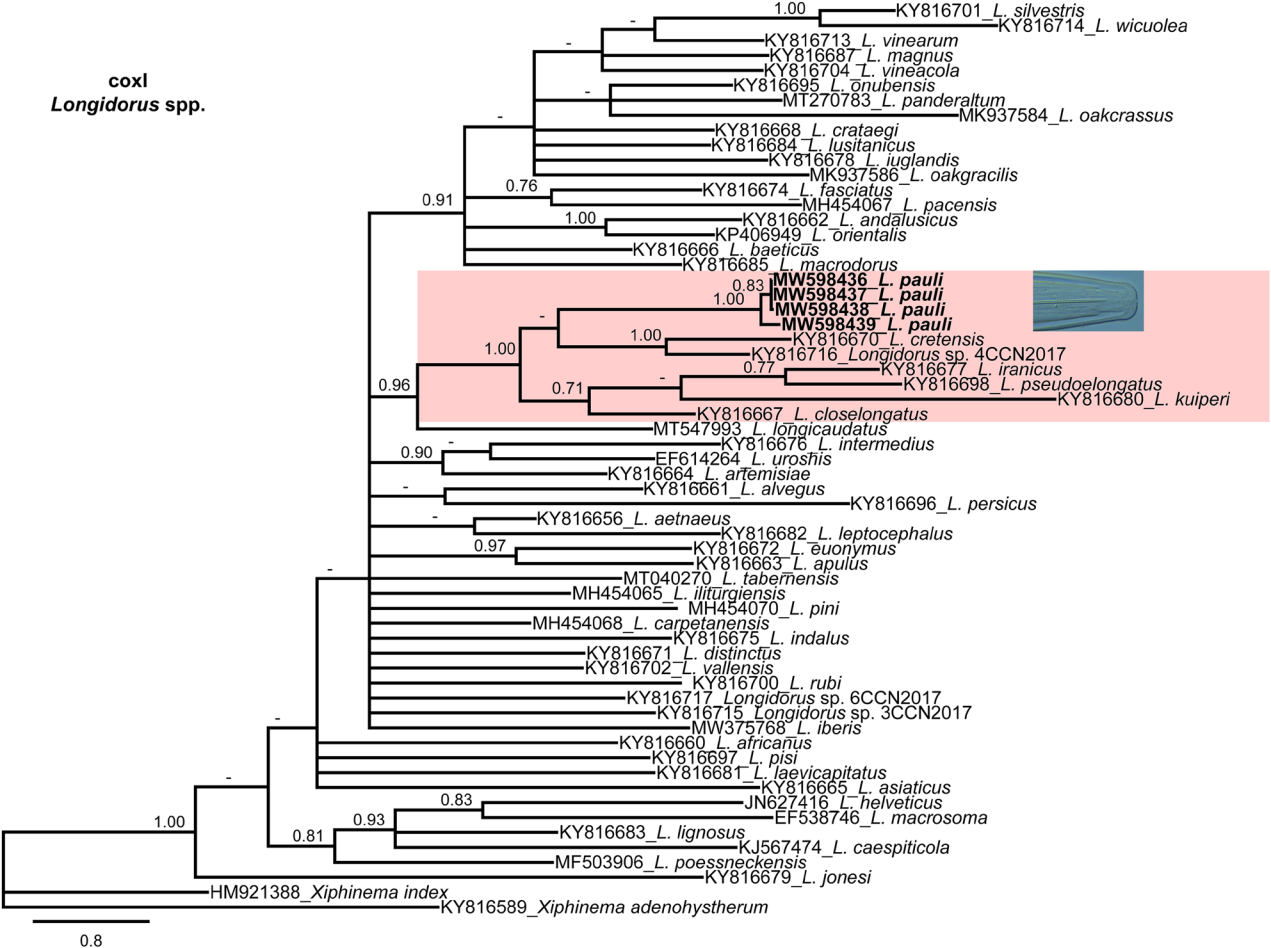
Phylogenetic relationships of *Longidorus pauli* (Lamberti et al., 1999) within the genus *Longidorus*. Bayesian 50% majority rule consensus trees as inferred from cytochrome c oxidase subunit I (coxI) mtDNA gene sequence alignments under the GTR + I + G model. Posterior probabilities more than 0.70 are given for appropriate clades. Newly obtained sequences in this study are in bold letters.

Phylogenetic relationships among *Longidorus* species, as inferred from analyses of D2-D3 expansion domains of 28S rRNA, ITS1, and the partial coxI mtDNA gene sequences using BI, are shown in Figures 2-4, respectively. The phylogenetic trees generated with the ribosomal and mitochondrial DNA markers included 109, 10 and 61 sequences with 749, 992 and 390 characters in length, respectively (Figs. 2-4). The D2-D3 region of the 28S rRNA tree of *Longidorus* spp. showed a well-supported subclade (PP = 1.00), including *L. pauli* (MW598384-MW598388), *L. iranicus*, *L. pseudoelongatus*, *L. proximus*, *Longidorus* sp. 4SAS2014 and *L. cretensis* (Fig. 2). *Longidorus pisi* from Thessaloniki clustered into a subclade with another *L. pisi* population from Iran (Fig. 2).

Due to scarce similarity with other sequences of the genus Longidorus, the phylogenetic reconstruction using the marker ITS1 sequences was difficult to obtain, therefore only related sequences were used for the phylogeny study. The 50% majority rule consensus ITS1 BI tree showed a low-supported clade (PP = 0.71) including *L. pauli* (MW598390-MW598392), *L. iranicus*, *L. pseudoelongatus*, and *L. cretensis* (Fig. 3). Finally, the phylogenetic relationships of *Longidorus* species inferred from analysis of the partial coxI gene sequences showed that *L. pauli* (MW598436-MW598439) clustered with the closed species in a well-supported clade (PP = 1.00) including *L. cretensis*, *Longidorus* sp. 4CCN2017, *L. iranicus*, *L. pseudoelongatus*, *L. kuiperi*, and *L. closelongatus* (Fig. 4).

Phylogenetic analyses based on three rDNA molecular markers (D2-D3 expansion domains of 28S rRNA gene and ITS1 region) and mitochondrial DNA coxI resulted in a general consensus of species phylogenetic positions for the majority, and were generally congruent with those given by previous phylogenetic analysis (Archidona-Yuste et al., 2019; Cai et al., 2020; Clavero-Camacho et al., 2021; Gutiérrez-Gutiérrez et al., 2013; Inserra et al., 2021). This research increased the number of *Longidorus* species in Greece, as well as the molecular diversity within *Longidorus*. In particular, phylogenetic results are congruent with morphological traits, since the new sequenced population of *L. pauli* clustered together with other species showing lip region rounded and set off by a slight depression from the rest of body, anteriorly slightly rounded to flattened, moderate long odontostyle, and tail conoid-rounded with bluntly rounded terminus.

In summary, the present study confirms the correct identity of this nematode and increase the great biodiversity of this genus in the Mediterranean Basin.
